# ETS-Related Gene Expression in Healthy Femoral Arteries With Focal Calcifications

**DOI:** 10.3389/fcell.2021.623782

**Published:** 2021-06-16

**Authors:** Francesco Vasuri, Sabrina Valente, Ilenia Motta, Alessio Degiovanni, Carmen Ciavarella, Gianandrea Pasquinelli

**Affiliations:** ^1^Pathology Unit, IRCCS, Azienda Ospedaliero-Universitaria di Bologna, Bologna, Italy; ^2^Experimental, Diagnostic and Specialty Medicine Department (DIMES), University of Bologna, Bologna, Italy

**Keywords:** ERG, EndMT, arterial calcification, elastic lamina, femoral artery

## Abstract

Bone development-related genes are enriched in healthy femoral arteries, which are more prone to calcification, as documented by the predominance of fibrocalcific plaques at the femoral location. We undertook a prospective histological study on the presence of calcifications in normal femoral arteries collected from donors. Since endothelial-to-mesenchymal transition (EndMT) participates in vascular remodeling, immunohistochemical (IHC) and molecular markers of EndMT and chondro-osteogenic differentiation were assessed. Transmission electron microscopy (TEM) was used to describe calcification at its inception. Two hundred and fourteen femoral arteries were enrolled. The mean age of the donors was 39.9 ± 12.9 years; male gender prevailed (M: 128). Histology showed a normal architecture; calcifications were found in 52 (24.3%) cases, without correlations with cardiovascular risk factors. Calcifications were seen on or just beneath the inner elastic lamina (IEL). At IHC, SLUG was increasingly expressed in the wall of focally calcified femoral arteries (FCFA). ETS-related gene (ERG), SLUG, CD44, and SOX-9 were positive in calcifications. RT-PCR showed increased levels of BPM-2, RUNX-2, alkaline phosphatase, and osteocalcin osteogenic transcripts and increased expression of the chondrogenic marker, SOX-9, in FCFA. TEM documented osteoblast-like cells adjacent to the IEL, releasing calcifying vesicles from the cell membrane. The vesicles were embedded in a proteoglycan-rich matrix and were entrapped in IEL fenestrations. In this study, ERG- and CD44-positive cell populations were found in the context of increased SLUG expression, thus supporting the participation of EndMT in FCFA; the increased transcript expression of osteochondrogenic markers, particularly SOX-9, reinforced the view that EndMT, osteochondrogenesis, and neoangiogenesis interact in the process of arterial calcification. Given its role as a transcription factor in the regulation of endothelial homeostasis, arterial ERG expression can be a clue of endothelial dysregulation and changes in IEL organization which can ultimately hinder calcifying vesicle diffusion through the IEL fenestrae. These results may have a broader implication for understanding arterial calcification within a disease context.

## Introduction

Focal arterial calcifications are a known but largely unreported entity, most often found in the muscular arteries of the extremities and occasionally of the viscera. Originally described in detail by Mönckeberg (1903), these focal calcifications have long been considered an age-related phenomenon. Yet, the causes, clinical significance, and fine morphology are not completely understood, and they are also not consistent in the literature. Micheletti et al. (2008) pointed out that this calcification pattern may be of help for understanding the process of vascular calcification in pathological conditions. Based on a retrospective study performed on 14 artery samples from different anatomical sites which did not include the femoral district, the authors concluded that focal calcifications involve a range of small muscular arteries with no or minimal histological inflammation. These calcification foci were seen both in the media and in the inner elastic lamina (IEL), but the authors hypothesized that the calcium deposition may begin on or just beneath the IEL (Micheletti et al., 2008).

The finding of calcifications in localized areas of otherwise healthy femoral arteries could reflect the increased expression in bone development-related genes, enriched in healthy femoral arteries (Steenman et al., 2018). In fact, femoral arteries are more prone to develop fibrocalcific plaques in a pathological context than other arterial peripheral districts (Herisson et al., 2011); furthermore, in this anatomical site, the atherosclerotic plaques are often associated with osteoid metaplasia (Sato et al., 2020) and higher expression of genes correlated to endochondral bone morphogenesis and endothelial-to-mesenchymal transition (EndMT), compared with pathological carotid arteries (Steenman et al., 2018).

Among the most recently studied EndMT mediators, the ETS-related gene (ERG) is the most expressed in arterial and venous endothelial cells (Hollenhorst et al., 2004), where it acts as a transcription factor for the control of endothelial homeostasis, activation, neoangiogenesis, and cell-to-cell as well as cell-to-matrix adhesion (Shah et al., 2016). On the other hand, ERG was observed to repress inflammatory cell activity and adhesion to the vessel wall *in vitro* by downregulating NF-kB, IL-8, and CD44, thus contributing to endothelial protection and stabilization (Yuan et al., 2009; Sperone et al., 2011). These results were recently confirmed by the finding that endothelial cells of regressing hyaloid vessels underwent downregulation of ERG and Friend leukemia integration 1 (FLI1), prior to apoptosis (Schafer et al., 2020).

A previous paper from our group reported a large histological series composed of 143 femoral artery segments from multi-organ donors with a mean age of 38 years. Our study showed that focal arterial calcification was present in 36 out of 143 (25.2%) cases (Vasuri et al., 2016).

The aims of the present study are to extend the characterization of the previous series of healthy femoral arteries and to assess the role of ERG and other mediators of EndMT in the pathogenesis of femoral focal calcifications, in order to better understand their relationship with calcified arterial disease.

## Materials and Methods

### Study Population and Histopathology

All femoral artery specimens sampled in the last 5 years from the Regional Cardiovascular Tissue Bank in Bologna were retrospectively evaluated, as previously described (Vasuri et al., 2016). Tissue comes from the femoral specimens of multi-organ donors, taken for histopathological evaluation of tissue suitability.

The following clinical data were assessed: age, gender, smoking habit, body mass index, hypertension, diabetes, hypercholesterolemia, obesity (body mass index ≥ 30), cardiopathy, pneumopathy, vasculopathy, and/or nephropathy.

At the time of the first diagnosis for tissue suitability, femoral artery samples were fixed in formalin, embedded in paraffin (FFPE), and routinely processed. Hematoxylin–eosin and trichrome stains were performed for histopathological analysis. The presence of intimal and/or medial calcifications was recorded.

### Immunohistochemistry

Three-micrometer-thick sections were cut from the tissue FFPE blocks. Immunohistochemistry (IHC) for ERG, factor VIII (FVIII), CD99, S100, and β-catenin was carried out with the automatic immunostainer Benchmark Ultra^®^ (Ventana Medical Systems, Roche Group, Tucson, AZ 85755, United States). The primary antibodies and the technical specifications are listed in Table 1. For the IHC analysis, we selected the 40 most recent cases [20 focally calcified femoral arteries (FCFA) and 20 not calcified femoral arteries (NCFA)] with comparable state of preservation and without previous application of decalcification protocols.

**TABLE 1:** Technical characteristics of the antibodies used in automated and manual IHC.

Antibody	Antigen retrieval	Clone	Dilution	Incubation Ab primary	Detection system
ERG	Ultra CC1 95°C 24 min	EPR3864	Prediluted	20 min at 36°C	OptiView DAB Detection Kit
CD99	Ultra CC1 95°C 32 min	O13	Prediluted	32 min at 36°C	OptiView DAB Detection Kit
S100	Ultra CC1 95°C 8 min	Polyclonal	Prediluted	12 min at 36°C	OptiView DAB Detection Kit
β-Cat	Ultra CC1 95°C 32 min	14	Prediluted	16 min at 36°C	OptiView DAB Detection Kit
CD44	Citrate buffer at pH = 6, autoclave 120°C 21 min	G44-26	1:100	o/n at 4°C	NovoLink^TM^ Polymer Detection System
SLUG	Citrate buffer at pH = 6, autoclave 120°C 21 min	A-7	1:500	o/n at 4°C	NovoLink^TM^ Polymer Detection System
SOX-9	Citrate buffer at pH = 6, autoclave 120°C 21 min	3C10	1:500	o/n at 4°C	NovoLink^TM^ Polymer Detection System
VEGF-R2	Citrate buffer at pH = 6, heat bath 98.5°C 20 min	D5B1	1:1,600	o/n at 4°C	NovoLink^TM^ Polymer Detection System

*o/n, overnight.*

IHC for CD44, vascular endothelial growth factor-receptor 2 (VEGF-R2), Snail Family Transcriptional Repressor 2 (SLUG or SNAI2), and sex-determining region Y box transcription factor-9 (SOX-9) was manually performed using a non-biotin-amplified method (NovoLink^TM^ Polymer Detection System; Leica, Newcastle upon Tyne, United Kingdom), according to the manufacturer’s instruction. Three-micrometer-thick sections obtained from FFPE blocks were dewaxed with xylol and rehydrated in graded ethanol. Antigenicity was retrieved using citrate buffer at pH 6, at 120°C, and 1 atm for 21 min for CD44, SOX-9, and SLUG antibodies and in a 20-min heat bath (98.5°C) for VEGF-R2, followed by cooling and rinsing in distilled water. After the neutralization of endogenous peroxidase activity in 3% H_2_O_2_ in absolute methanol in the dark for 5 min at room temperature, samples were labeled with CD44 (1:100; BD Biosciences Pharmingen, San Jose, CA, United States), VEGF-R2 (1:1,600; D5B1, Cell Signaling, Danvers, MA, United States), SLUG (1:500; Santa Cruz Biotechnology, Dallas, TX, United States), and SOX-9 (1:500; Abcam, Cambridge, MA, United States) primary antibodies diluted in 1% BSA in PBS o/n at 4°C in a wet chamber. Primary antibodies were omitted in negative controls. Then, sections were exposed to 3,3′-diaminobenzidine (DAB) substrate/chromogen, counterstained with hematoxylin, dehydrated in a series of graded ethanol, and coverslipped.

Tissue slides were observed with a Leitz Diaplan light microscope (Wetzlar, Germany); digital images were acquired using Image-Pro Plus 6 software (Media Cybernetics, Rockville, MD, United States) at 10 × for VEGFR2 and SOX-9 and 25 × for SLUG. To quantify VEGF-R2, SOX-9, and SLUG positive areas, five random digital images taken from each sample were analyzed using ImageJ Fuji distribution (Schindelin et al., 2012). Results are expressed as percentage of positive area/total area.

### Transmission Electron Microscopy

For electron microscopy, small femoral arterial samples were fixed in 2.5% glutaraldehyde in cacodylate buffer 0.1 mol/L pH 7.4, for 4 h. The samples were then stored in cacodylate buffer at 4°C. Tissue was then postfixed with a solution of 1% osmium tetroxide in 0.1 mol/L cacodylate buffer and embedded in araldite after a graded alcohol serial dehydration step. Semithin sections were stained with toluidine blue and observed at light microscopy to identify the areas of calcification. Sixty- to eighty-nm thin sections were stained by uranyl acetate and lead citrate and then observed in a transmission electron microscope CM100 Philips (Thermo Fisher, Waltham, MA, United States) at an accelerating voltage of 80 kV. Images were recorded with a MegaView digital camera.

### RNA Extraction and Quantitative Real-Time Analysis

RT-PCR analysis was carried out on 20 cases (10 FCFA and 10 NCFA), selected according to temporal and preservation criteria. Total RNA was extracted from FFPE tissues using RecoverAll^TM^ Total Nucleic Acid Isolation Kit (Invitrogen, Carlsbad, CA, United States), according to the manufacturer’s instructions, with overall yields ranging from 1.4 to 9.8 ng/μl. Reverse transcription was performed from 1.5 μl of total RNA in 7.5 μl reaction volume using High Capacity Reverse Transcription Kit (Life Technologies, Carlsbad, CA, United States) and specific primers (Table 1). Primers specific for the target genes were designed using the NCBI BLAST tool (purchased from Merck, Kenilworth, NJ, United States; Table 2). Real-time PCR was carried out in a CFX Connect Real-Time PCR Detection System (Bio-Rad, Hercules, CA, United States) using the SYBR green mix (Bio-Rad). Each assay was performed in triplicate and target gene expression was normalized to the housekeeping gene glyceraldehyde 3-phospate dehydrogenase (*GAPDH*). Final results were determined by the comparative 2^−^ΔΔ*Ct* method and expressed as fold changes relative to controls (Livak and Schmittgen, 2001).

**TABLE 2:** List of primer sequences used for reverse transcription and real-time PCR.

Gene name	Primer sequences
GAPDH	FWD AATGGGCAGCCGTTAGGAAA REV AGGAGAAATCGGGCCAGCTA
BMP-2	FWD TGTCTTCTAGCGTTGCTGCT REV CAACTCGAACTCGCTCAGGA
RUNX-2	FWD TGATGACACTGCCACCTCTGA REV GCACCTGCCTGGCTCTTCT
SOX-9	FWD AGTACCCGCACCTGCACAAC REV CGCTTCTCGCTCTCGTTCAG
ALP	FWD GGGCTCCAGAAGCTCAACAC REV GTGGAGCTGACCCTTGAGCAT
OCN	FWD CACCGAGACACCATGAGAGC REV CTGCTTGGACAAAGGCTGC

### Statistical Analysis

GraphPad Prism software was used for all statistical analyses. Variables are expressed as means ± standard deviations, ranges, and frequencies. The unpaired *t*-test was used when appropriate. A *p*-value ≤ 0.05 was considered as significant.

## Results

### Study Population and Histopathology

This is a retrospective monocentric study on archival tissue from multi-organ deceased donors. In this population, the anonymous treatment of data, conducted according to the Ethical Guidelines of the 1975 Declaration of Helsinki and following revisions, is permitted by the Privacy Representative, according to Italian Law (G. U. Repubblica Italiana, No 72, 26/03/20120). No consent was therefore required.

We reviewed 214 femoral artery specimens from 128 (59.8%) male and 86 (40.2%) female multi-organ donors, with a mean age of 39.9 ± 12.9 years (range 14–60 years). Smoke habit was recorded in 61 (28.5%) donors, cardiopathy in 10 (4.7%), pneumopathy in 6 (2.8%), hypertension in 22 (10.3%), diabetes in 5 (2.3%), dyslipidemia in 7 (3.3%), nephropathy in 5 (2.3%), and vasculopathy in 3 (1.4%). A significant association with the presence of arterial calcifications was observed for none of these risk factors.

**FIGURE 1:**
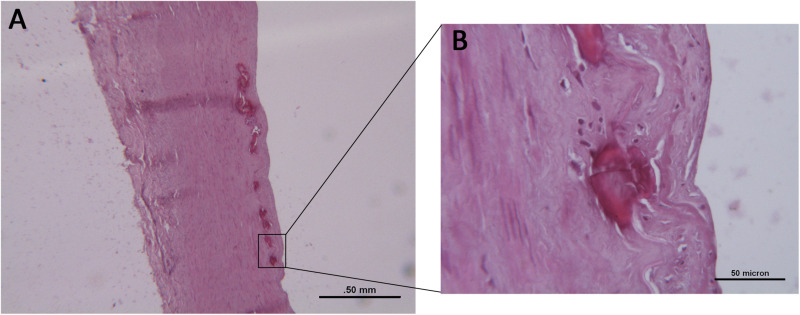
**(A)** Histological examination of a focally calcified femoral artery (FCFA) with preserved architecture and multiple calcifications of the inner elastic lamina. Hematoxylin–eosin stain. Scale bar = 0.5 mm. **(B)** At higher magnification, discontinuity and fragmentation of the elastic lamina are evident. Scale bar = 50 μm.

Histologically, the femoral arteries were morphologically normal; however, multiple foci of calcification were observed in 52 (24.3%) cases; these cases were labeled as FCFA (Figure 1A); the other cases were indicated as NCFA. Among FCFA, 12 focal calcifications were localized in the media layer, 21 in the intima/IEL, and in 19 cases, they were present in both layers. When calcium deposits involved IEL, discontinuation and rupture of the elastic fibers was seen (Figure 1B). Overall, these results overlapped with those from our previous report; in particular, mean donors’ age, gender distribution, correlation with cardiovascular risk factors, incidence of focal calcifications, and their distribution in the arterial wall were similar (Vasuri et al., 2016).

### Expression of Early EndMT and Chondrogenic Markers in the Femoral Arterial Wall

**FIGURE 2:**
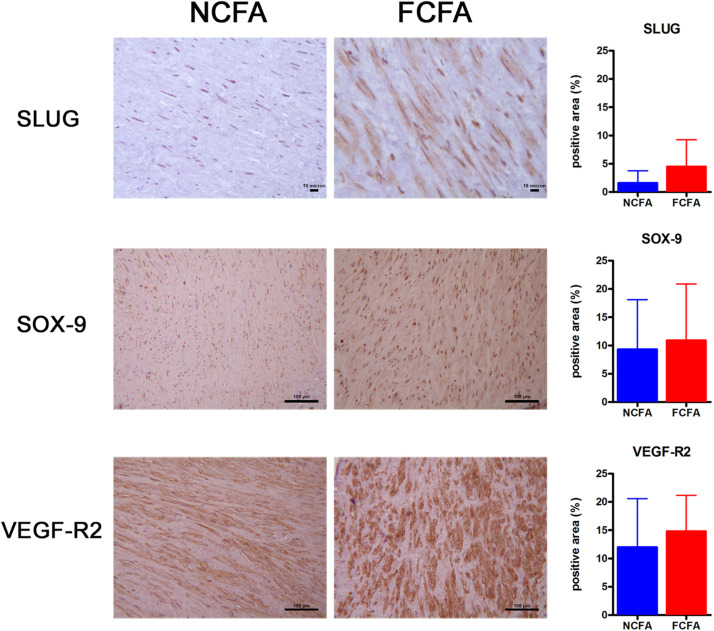
The immunohistochemical analysis of the entire arterial wall showed increased positivity for SLUG, a marker of EndMT, in the focally calcified femoral arteries (FCFA, *n* = 20) (*p* = 0.0006). Positivities observed for SOX-9 and VEGF-R2 showed no significant change between FCFA and not calcified femoral arteries (NCFA, *n* = 20), although the FCFA showed a trend of greater expression. Scale bar for SLUG = 10 μm; scale bar for SOX-9 and VEGF-R2 = 100 μm.

The first part of the *in situ* IHC study consisted in the observation of the investigated markers on the entire section of the arterial wall (Figure 2), thus focusing on the smooth muscle cell layer.

SLUG was significantly more expressed in FCFA than in NCFA, with a mean of 4.51 ± 0.75 and 1.62 ± 0.33 (positive area/total area ratio), respectively (*p* = 0.0006, *t*-test). VEGF-R2 showed a trend of higher expression in FCFA than in NCFA, with a mean positive area/total area ratio of 14.83 ± 1.03 and 12.02 ± 1.25, respectively (*p* = 0.095). SOX-9 was only slightly more expressed in FCFA, with a mean positive area/total area ratio of 10.90 ± 1.62 and 9.33 ± 1.34 in FCFA and NCFA, respectively (*p* = 0.455).

Except for the markers reported above, the others were only qualitatively assessed since we did not find positive smooth muscle cells in FCFA nor in NCFA. S100 was positive exclusively in small adventitial nerves, while CD99 was positive in the *vasa vasorum* and mesenchymal adventitial cells; FVIII and β-catenin were expressed primarily in luminal endothelial cells and *vasa vasorum*.

### Expression of Early EndMT and Chondrogenic Markers in Focal Arterial Calcification

**FIGURE 3:**
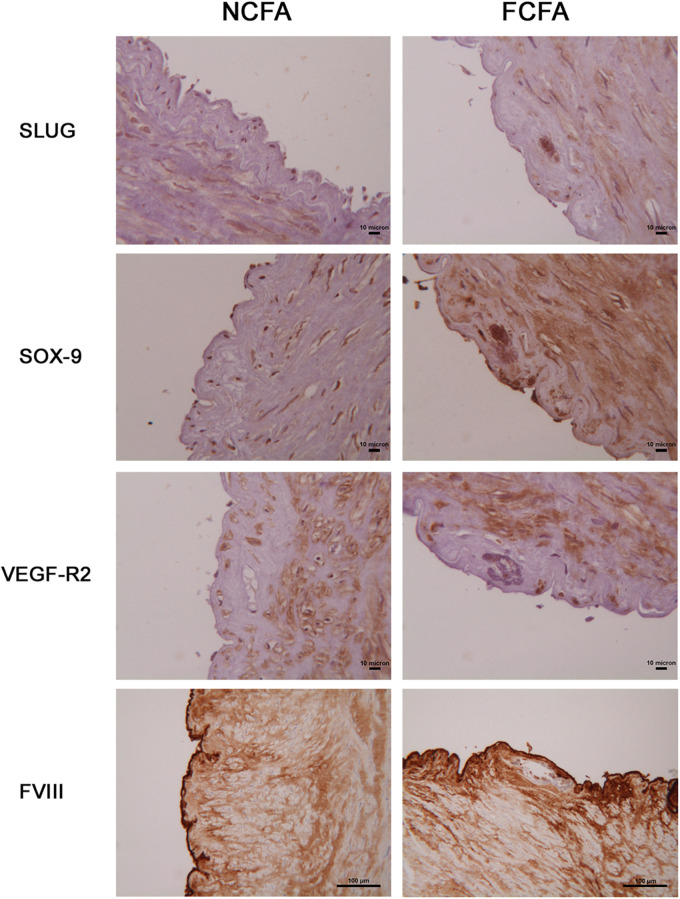
Immunohistochemical analysis of the areas of calcification showed marked SLUG and SOX-9 positivity; in contrast, the endothelial cell lineage marker, FVIII, and the endothelial cell receptor, VEGF-R2, were negative. Scale bar for SLUG, SOX-9, and VEGF-R2 = 10 μm; scale bar for FVIII = 100 μm.

**FIGURE 4:**
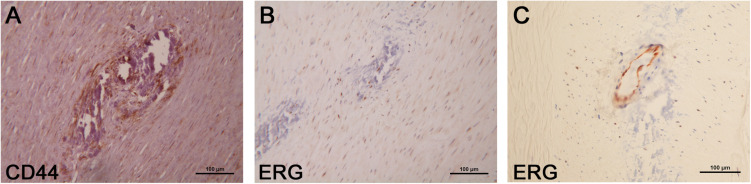
Additional immunohistochemical analysis of the areas of calcification showed CD44 **(A)** and ERG **(B)** positivity within and at the periphery of the calcifications where these markers were expressed by mesenchymal spindle cells; ERG was also positive in the endothelial cells that line the newly formed vessels present within the calcifications **(C)**. Scale bar of all images = 100 μm.

We analyzed the expression of some of the above-reported markers in the foci of calcification. As schematized in Figure 3, SLUG and SOX-9 were intensively positive; furthermore, CD44 and ERG were expressed in spindle mesenchymal cells seen within and at the periphery of the calcifications (Figure 4); interestingly, ERG stained newly formed vessels within the foci of calcification. In NCFA, CD44 and ERG were expressed in the *vasa vasorum* and luminal endothelial cells exclusively.

### Expression of Osteochondrogenic Transcripts in the Femoral Arterial Wall

**FIGURE 5:**
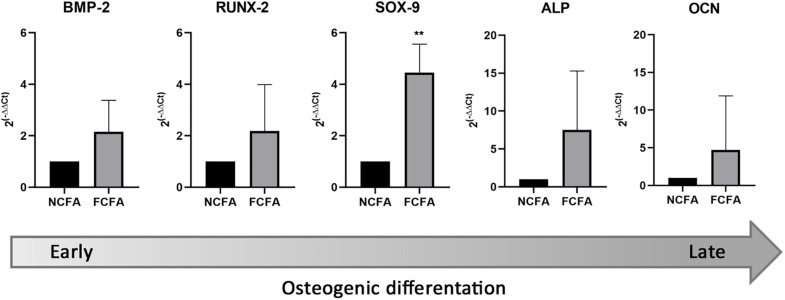
Focally calcified femoral arteries (FCFA, *n* = 10) showed an overall increase of transcripts involved in early and late osteochondrogenesis compared with not calcified femoral arteries (NCFA, *n* = 10); however, the expression gradient was only significant for SOX-9 (**p = 0.0033).

The expression of osteochondrogenic transcripts was performed on FFPE sections therefore giving an average transcript representation of the whole femoral arterial wall. Except for SOX-9, we did not find any significant fold change in transcript expression; however, FCFA had an overall tendency to express more transcripts related to the osteochondrogenic differentiation program than NCFA (Figure 5). The mean fold changes of the investigated transcripts in the FCFA compared with NCFA were as follows: 2.16 ± 1.22 for BMP-2, 2.18 ± 1.81 for RUNX-2, 7.53 ± 7.76 for ALP, 4.45 ± 1.11 for SOX-9, and 4.71 ± 7.19 for OCN. The only transcript significantly higher in FCFA than in NCFA was SOX-9 (*p* = 0.0033).

### Transmission Electron Microscopy

**FIGURE 6:**
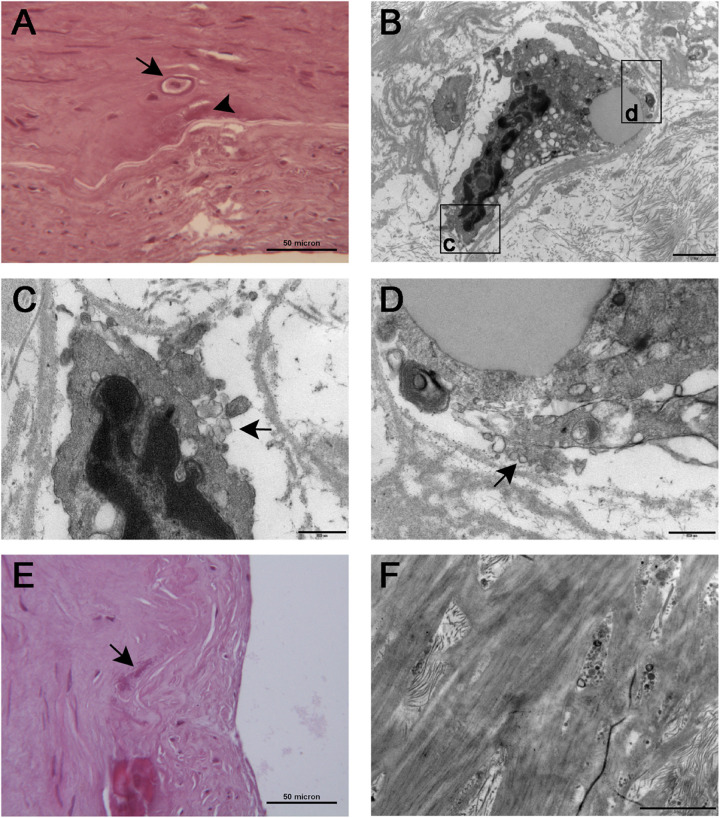
Histological **(A)** and TEM **(B)** appearance of osteoblast-like cells [arrow in **(A)**] seen in proximity of the inner elastic lamina. The cells were embedded in an empty lacuna that ultrastructural examination showed to be rich in particulate and filamentous proteoglycans. The boxed areas are seen at higher magnification in **(C,D)**, respectively; cell membrane ruffling with extrusions and matrix vesicles budding from the cell surface (arrows) can be appreciated in both images. **(E)** At histology, large aggregates of calcified vesicles were seen as extracellular deposition of amorphous, basophilic substance [arrow in **(E)**, also seen in **(A)**, arrowhead] close to the inner elastic lamina. **(F)** TEM showed clusters of calcified matrix vesicles permeating the fenestrations of the inner elastic lamina. Scale bars = 50 μm **(A,E)**, 2 μm **(B,F)**, and 0.5 μm **(C,D)**.

TEM showed osteoblastic-like cells (Figure 6B) close to the IEL; these cells, after careful research, were also seen at histology where they appeared embedded in an empty lacuna (Figure 6A) which electron microscopy showed to be made up of collagen fibers and particulate and filamentous proteoglycans; at higher magnification, clusters of extracellular vesicles (50–200 nm in diameter) were seen at the plasma cell membrane and free in the matrix (Figures 6C,D); here, the vesicles were concentrated on the surface of the IEL and permeated its fenestrations (Figure 6F). Some of the vesicles were markedly electron dense, acting as a site of calcium deposition. On histology, large aggregates of calcified vesicles were seen as extracellular deposition of amorphous, basophilic substance at the IEL (Figures 6A,E).

## Discussion

The mechanism of arterial calcification is still largely unknown. Different pathogenetic mechanisms are reported to be involved: genetic factors as in the model of *pseudoxantoma elasticum* (D’Marco et al., 2020), epigenetic factors fueling atherosclerosis (Kwon et al., 2020), changes in transcriptional and post-transcriptional regulation control, as miRNA–mRNA axis (Vasuri et al., 2020), injuries leading to vascular wall necrosis and inflammation, differentiation of resident or circulating vascular progenitors (Psaltis et al., 2014; Vasuri et al., 2014), metabolic factors (Demer and Tintut, 2014), and local context factors. This heterogeneity may account for the presence of different vascular calcification disease morphologies and phenotypes.

The present study confirms our previous experience (Vasuri et al., 2016) that calcification affects even normal femoral arteries focally; this is a condition more frequent than expected. In a series of 214 consecutive femoral arteries, focal calcifications affected 24% of a young population whose arteries underwent histological analysis for establishing the suitability for arterial transplant programs. Likewise, to a previous report, their presence did not show any significant correlation with major known cardiovascular risk factors nor with minor morphological arterial changes (Micheletti et al., 2008). Males are affected more than females and the calcified arteries belong, structurally, to the muscular arterial category. In the previously reported series, the involved vessels were the *dorsalis pedis*, *posterior tibialis*, radial and temporal arteries, and small arteries of the thyroid, breast, and uterus (Micheletti et al., 2008). This confirms that muscular arteries have a predilection for developing calcifications under normal condition probably due to their increased expression in bone development-related genes (Steenman et al., 2018).

As already hypothesized in atherosclerosis (Chen et al., 2020), EndMT could be involved in this process. In fact, we found an increased immunoreactivity for SLUG by analyzing the entire thickness of the FCFA vascular wall, along with an intense immunostaining for markers related to EndMT (SLUG and ERG), to stemness (CD44), and to chondrogenesis (SOX-9), in correspondence to the areas of calcification. This pattern of positivity suggests a link between EndMT and osteochondrogenic differentiation which is also reported in the literature. In fact, SLUG, a member of the SNAIL family of transcriptional repressors, is known to have a role as an early regulator of epithelial cell fate and lineage commitment (Phillips and Kuperwasser, 2014). ERG, the ETS-related gene, is a transcription factor involved in the regulation of endothelial and cartilage homeostasis; ERG also regulates angiogenesis and stabilizes the endothelium maintaining junction integrity through its transcriptional regulation of junctional molecules (Shah et al., 2016). In combination with other cell surface molecules, the surface receptor CD44 is a common marker for both normal and cancer stem cells, acting as a receptor for hyaluronan, the major component of stem cell niches (Skandalis et al., 2019); however, CD44 is also expressed by inflammatory cells and even by endothelial cells (Trochon et al., 1996). CD44 was also found to be important for epithelial–mesenchymal transition induction by TGF-β (Kolliopoulos et al., 2018). Interestingly, a population of CD44^+^ multipotent mesenchymal stem cells has been found within the adult human arterial adventitia with properties of differentiation into adipocytes, chondrocytes, and osteocytes under culture conditions (Klein et al., 2011). This observation opens the question about the role that such cell population can have in arterial calcification. SOX-9 is a transcription factor regulating many morphogenetic processes; it is highly expressed in normal cartilage cells where it regulates proliferation and extracellular matrix production during chondrogenesis (Rockich et al., 2013), but it is also expressed in tumors of cartilage lineage (Wehrli et al., 2003).

In this study, at least two cell components were seen involved in the calcification process: CD44-positive mesenchymal cells that electron microscopy has seen to be placed within a proteoglycan-rich lacuna; these cells had an osteoblastic commitment and exhibited extensive cell membrane ruffling, with abundant extrusions and matrix vesicles budding from the cell surface; the latter finding is consistent with osteogenic cells actively promoting mineralization in the surrounding matrix (Zazzeroni et al., 2018). This mechanism is also similar to that described in physiological and pathological ossification of human tissues, where the matrix calcification is mediated by alkaline phosphatase-containing vesicles acting as site of apatite crystal accumulation (Golub, 2009; Bonucci, 2013). Indeed, we observed the presence of ERG-positive mesenchymal cells, possibly deriving from EndMT of the luminal endothelium and giving origin to neovessels adjacent to and within the intimal calcification. The connection between osteochondrogenesis and neoangiogenesis is well known (Cocks et al., 2017).

The presence of ERG^+^ and CD44^+^ cell populations, in a context of increased SLUG expression, supports the involvement of EndMT in FCFA; moreover, the increased transcript expression of osteochondrogenic markers, particularly SOX-9, reinforces the suggestion that EndMT, osteochondrogenesis, and neoangiogenesis are involved in an apparent context of morphological arterial normality. On the other hand, β-catenin expression did not change significantly, albeit it was expected to be altered, since it is described to be involved in EndMT and to stimulate chondrogenesis by increasing SOX-9 expression (Kirton et al., 2007). Supporting the chondrogenesis involvement, the SOX-9 transcript was significantly increased in FCFA samples, while calcifications were intensely SOX-9 positive at IHC. In addition, the ultrastructural examination of the matrix adjacent to the area of calcification revealed a particular richness in particulate and filamentous proteoglycans, one of the characteristics of the chondrogenic matrix. Furthermore, a similar matrix rich in hyaluronan and versicans was associated with a synthetic smooth muscle phenotype under PDGF and TGF-β stimulation; this provisional matrix facilitates migratory cell activities and angiogenesis (Wight, 2017). A recent *in vitro* study showed that hyperglycemia-induced exosomal vesicles from HUVEC are able to promote calcification in vascular smooth muscle cells *via* versican activation (Li et al., 2019). Therefore, the proteoglycan-rich matrix has an effect on vascular cell mobility, shape change, and differentiation.

Our study confirms that in muscular arteries the calcification rises in close proximity to IEL; this preferential location, also because there are few studies on this component of the vascular matrix, remains unexplained. However, the 3D architecture of IEL is more complex of what expected, as can be supposed from the studies performed on NaOH digested arteries and examined by scanning electron microscopy (Roach and Song, 1988). Also, IEL function in muscular resistance arteries is rather unclear; it is thought that in such arterial district changes in IEL, fenestrae size and number may alter diffusion of vasoactive substances between endothelial cells and smooth muscle cells leading to altered arterial responses (Halabi and Kozel, 2020). Since it was recently demonstrated that, unlike previously thought, in muscular arteries IEL synthesis is driven by the endothelium (Lin et al., 2019), it can be supposed that, in the context of EndMT, the synthesis of IEL may be altered as a consequence of luminal endothelial cell dysregulation and directed toward a morphology facilitating the entrapment of matrix vesicles within.

A limitation of the present study is that we studied a small arterial area in what appeared to be an otherwise normal arterial wall; this means that while morphology and IHC give reliable information on the differences between the calcified focus and the remaining portion of the artery, the expression analysis of the EndMT and osteochondrogenic genes could be not representative of the analyzed processes. Moreover, also the use of archival FFPE tissue can be not always reliable.

To our knowledge, this is the first study on the expression of ERG and other EndMT and osteochondrogenic markers in a series of normal human femoral arteries with foci on IEL calcification. Using immunohistochemistry, we documented ERG^+^ and CD44^+^ cell populations in the context of increased SLUG expression, thus supporting the participation of EndMT in FCFA; furthermore, the increased transcript expression of osteochondrogenic markers, particularly SOX-9, reinforced the view that EndMT, osteochondrogenesis, and neoangiogenesis interact in the process of focal calcification. Given its role as a transcription factor in the regulation of endothelial homeostasis, parietal ERG expression can be a clue of endothelial dysregulation and changes in IEL organization which can ultimately hinder matrix vesicle diffusion through the IEL fenestrae. These results are supposed to have a broader implication for understanding arterial calcification even in a disease context (Yuan et al., 2009; Sperone et al., 2011; Shah et al., 2016).

## Data Availability Statement

The raw data supporting the conclusions of this article will be made available by the authors, without undue reservation.

## Ethics Statement

Ethical review and approval was not required for the study on human participants in accordance with the local legislation and institutional requirements. Written informed consent for participation was not required for this study in accordance with the national legislation and the institutional requirements.

## Author Contributions

FV, SV, and GP: manuscript drafting. FV and SV: study design. FV: histology and immunohistochemistry analysis. FV, IM, and CC: data analysis. SV: TEM analysis. IM and CC: RT-PCR analysis. AD: tissue processing and immunohisotchemistry, and quality control. GP: study conceptualization and design. All authors revised the final draft and agreed with the submitted version of the manuscript.

## Conflict of Interest

The authors declare that the research was conducted in the absence of any commercial or financial relationships that could be construed as a potential conflict of interest.

## Abbreviations

ALP: alkaline phosphatase
DAB: 3,3 ′ -diaminobenzidine
EndMT: endothelial-to-mesenchymal transition
ERG: ETS-related gene
FCFA: focally calcified femoral artery
FFPE: formalin-fixed paraffin-embedded
FLI1: Friend leukemia integration 1
FVIII: factor VIII
GAPDH: glyceraldehyde 3-phospate dehydrogenase
IHC: immunohistochemistry
IEL: inner elastic lamina
BMP-2: bone morphogenetic protein-2
NCFA: not calcified femoral artery
OCN: osteocalcin
RUNX-2: runt-related transcription factor-2
SLUG: Snail Family Transcriptional Repressor 2
SOX-9: sex-determining region Y box transcription factor-9
TEM: transmission electron microscopy
VEGF-R2: vascular endothelial growth factor-receptor 2.
